# Supplementary material for: A unified evolutionary origin for the ubiquitous protein transporters SecY and YidC
Source: BMC Biol. 2021 Dec 15;19:266. doi: 10.1186/s12915-021-01171-5 (PMC8675477; doi:10.1186/s12915-021-01171-5)
Supplement: Supplementary file 1 — Additional file 1. Ancestral SecY sequence reconstruction [158–168]. [file 12915_2021_1171_MOESM1_ESM.pdf]

Ancestral SecY sequence reconstruction

We sought to estimate the amino acid sequences of cenancestral SecY and proto-SecY. A recent study of another internally duplicated cenancestral protein, the helix-hairpin-helix $(HhH)_2$, demonstrated that maximum likelihood (ML) tree inference using IQ-TREE (1) and empirical Bayesian ancestral sequence reconstruction (ASR) could yield a cenancestral sequence with dramatically increased sequence identity between its duplicated domains (from 21 ± 8% to 46%; (2)). Here we apply similar methods to a recently published dataset of taxonomically diverse prokaryotic SecY sequences (3). The published alignment of these sequences is inconsistent with a structural alignment around C.H1 (Figure S6), but aligning the sequences using MAFFT L-INS-i yields results consistent with the structures, so we use this realignment for our analysis. A few sequences were added or removed to obtain a final full-length (FL) alignment, as described in the Methods. In addition to the FL alignment, we also prepared a structure-guided alignment of the N- and C-half subsequences from the same dataset, as described in the Methods, and used this NC alignment as a separate basis for ASR. Finally, we also used a version of the NC alignment which includes only the five structurally aligned blocks corresponding to H1-5 (NC-blocks).

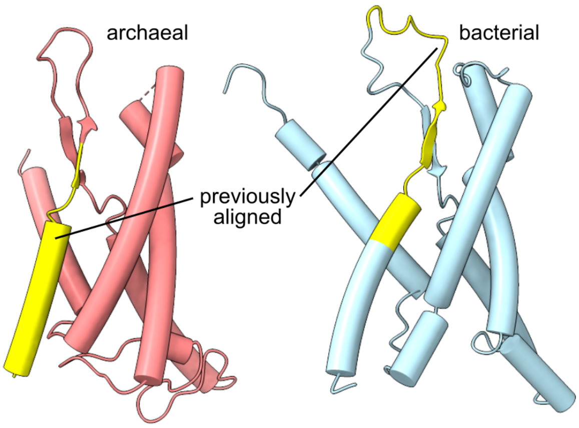

Figure S6. Discrepancy between a structural alignment of archaeal and bacterial C.H1 and the previous sequence alignment of Harris and Goldman, 2021. Archaeal (M. jannaschii, 1rh5) and bacterial SecY (G. thermodenitrificans, 6itc) C-halves are shown, with a previously sequence-aligned segment highlighted in yellow.

Matched-pair tests (4) did not reject the standard assumptions of symmetry and homogeneity ($p >$ 5%), although the data in NC-blocks were insufficient for most tests. Automated phylogenetic model selection was performed with ModelFinder (5) by the Bayesian information criterion (BIC). In each case, the same model would have been selected by the Akaike information criterion (AIC), whereas the corrected AIC (AICc), which more strongly avoids overfitting, would select a less parameter-rich model. Likelihood mapping (6) showed that while the phylogenetic signal in all three alignments is poor, it is strongest in the FL alignment, and not much degraded by reducing NC to NC-blocks. ML tree inference with IQ-TREE 2 (7) was performed with 1000 ultrafast bootstrap replicates (8).

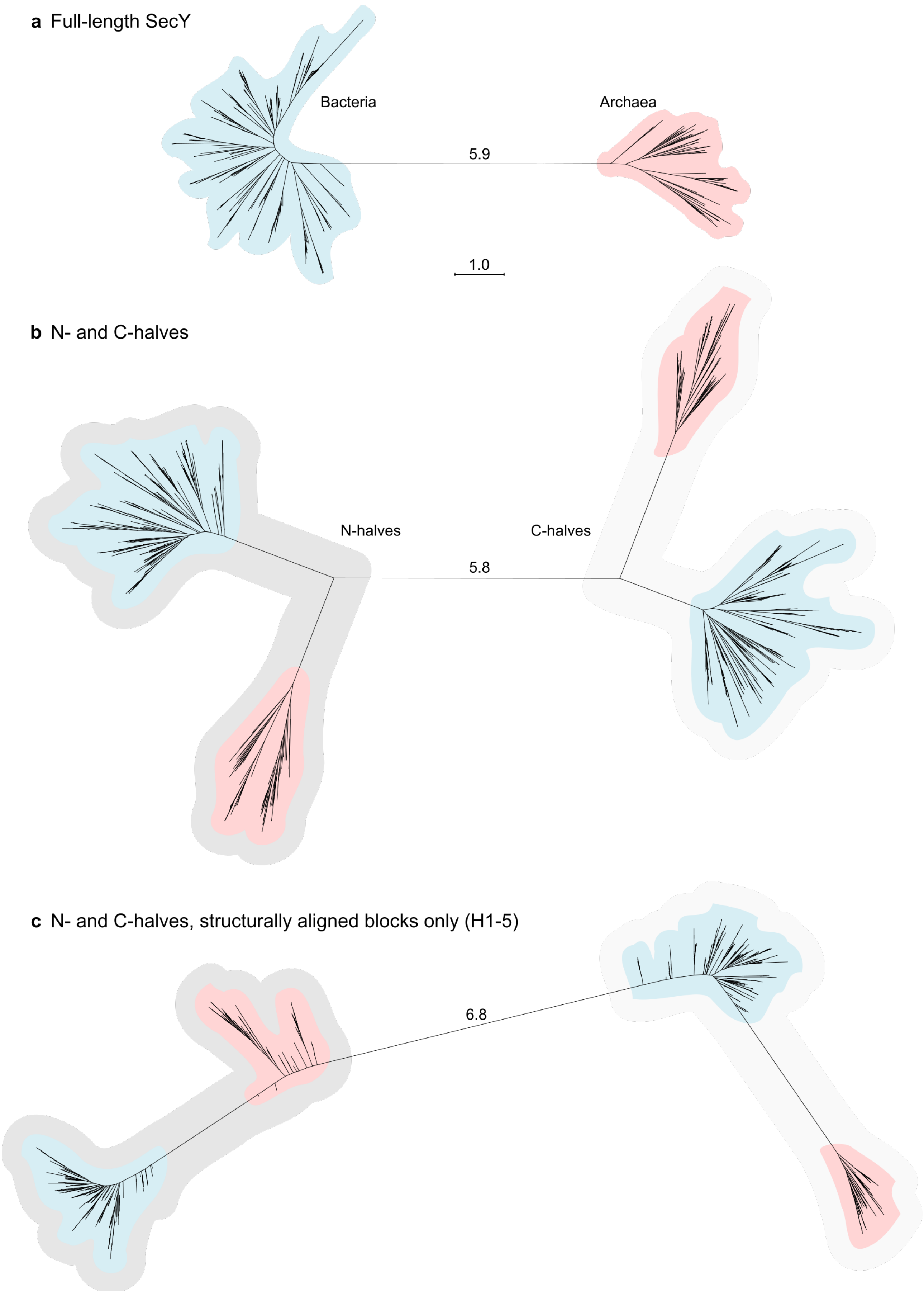

Figure S7. Maximum likelihood trees inferred for SecY and its halves. All trees are shown on the same scale. The length unit is the expected number of substitutions per site. a Tree inferred from the FL alignment. b Tree inferred from the NC alignment. c Tree inferred from the NC-blocks alignment.

The FL tree accurately reflects most of the shallow relationships within phyla, as in Harris & Goldman 2021. But the deepest branch, between archaea and bacteria, is extraordinarily long, 5.9 expected substitutions per site (sps), which means the overwhelming majority of sites should be expected to differ in both archaea and bacteria from whatever their identity was in the cenancestor (Figure S7a). This length may be regarded as a lower bound on the true branch length, since excluding the fastest-evolving sites or using more parameter-rich models typically yields even longer archaeal-bacterial branches (9). It is thus unsurprising that ancestral sequences reconstructed at the root nodes of archaea or bacteria, using IQ-TREE 2's implementation of the empirical Bayesian method, displayed halves with poor sequence similarity (15-20% identity across H1-5), not significantly different from that between the halves of extant sequences (16 ± 3% across H1-5).

The NC and NC-blocks alignments yielded trees with a long central branch separating the N-halves and C-halves (5.8, 6.8 sps; Figure S7b,c). The subtree for each of the two halves displayed some topological discrepancies with the other half's subtree. These discrepancies were particularly acute in the NC-blocks tree, which puts the root of the N-half inside archaea, but the root of the C-half inside bacteria. Such extreme discrepancies are common in trees of universal paralogs, which are generally so divergent that outgroup rooting is inadvisable due to severe model violations (10).

	**FL**	**NC**	**NC-blocks**
% gaps	51	67	0
**sites**	914	678	102
parsimony-informative	630	423	102
singleton	118	104	0
constant	166	151	0
**sequences**	342	684	684
composition χ^2 test $p < 5\%$	9	10	2
**matched pair test *p***			
symmetry	0.23	0.56	0.62
marginal symmetry	0.32	0.88	n/a
internal symmetry	0.26	0.34	n/a
**model selection**			
BIC	LG+F+R9	LG+F+R10	Q.yeast+F+R9
AIC	LG+F+R9	LG+F+R10	Q.yeast+F+R9
AICc	LG+F+R8	LG+G4	LG
**likelihood mapping**			
% fully resolved	66	55	52
% partially resolved	22	3	3
% unresolved	12	42	45
total tree length	313	147	163

Table S1. Summary statistics from sequence alignment and phylogenetic inference. Abbreviations: LG, Le & Gascuel substitution matrix (160); F, empirical amino acid frequencies; R, rate categories; G, gamma-distributed rate categories; Q.yeast, QMaker yeast-specific substitution matrix (161).

Unlike the NC-blocks tree, the NC tree places the root of both subtrees between archaea and bacteria. This difference is difficult to attribute to any meaningful signal in the data, since the NC-blocks alignment retains the high-confidence columns from structural alignment and nearly as much phylogenetic signal as the NC alignment (Table S1). It may instead be an artefact of long branch attraction (LBA), induced by the greater number of high variability and poorly aligned sites contained in the NC alignment. The indistinguishability of this tree topology from what LBA would induce makes the accuracy of universal paralog trees suspect in general (10). Thus although one

could estimate ancestral N- and C-half and proto-SecY sequences using these trees, one has little reason to think those estimates would be accurate.

The methods applied here may have proven less effective than when previously applied to HhH domains (2) because those domains are less divergent than the SecY halves. The HhH domains each conserve independent nucleotide-binding activity, and thus are just as conserved as are the $(HhH)_2$ proteins as a whole (22.4 ± 7.8% between domains *vs* 19.5 ± 4.4% between UvrC and ComEA). By contrast, the SecY domains form an obligate complex around a single active site, and are much less conserved than SecY as a whole (12.5 ± 2.2% between domains *vs* 20.3 ± 1.6% between archaea and bacteria). It is also known that general-purpose substitution models like LG, used here, are a poor fit to substitutions occurring in the heterogeneous membrane environment (11). Regardless of the true cause, the sequences of SecY's ancestors remain recondite. The main text therefore focuses on more stable characteristics, namely structure, mechanism, function, and a few functionally important, highly conserved residues.

References

1. Trifinopoulos J, Nguyen L-T, von Haeseler A, Minh BQ. W-IQ-TREE: a fast online phylogenetic tool for maximum likelihood analysis. Nucleic Acids Research. 2016 Jul 8;44(W1):W232–5.
2. Longo LM, Despotović D, Weil-Ktorza O, Walker MJ, Jabłońska J, Fridmann-Sirkis Y, et al. Primordial emergence of a nucleic acid-binding protein via phase separation and statistical ornithine-to-arginine conversion. PNAS. 2020 Jul 7;117(27):15731–9.
3. Harris AJ, Goldman AD. The very early evolution of protein translocation across membranes. PLoS computational biology. 2021;17(3):e1008623.
4. Naser-Khdour S, Minh BQ, Zhang W, Stone EA, Lanfear R. The Prevalence and Impact of Model Violations in Phylogenetic Analysis. Genome Biology and Evolution. 2019 Dec 1;11(12):3341–52.
5. Kalyaanamoorthy S, Minh BQ, Wong TKF, von Haeseler A, Jermiin LS. ModelFinder: fast model selection for accurate phylogenetic estimates. Nat Methods. 2017 Jun;14(6):587–9.
6. Strimmer K, von Haeseler A. Likelihood-mapping: A simple method to visualize phylogenetic content of a sequence alignment. PNAS. 1997 Jun 24;94(13):6815–9.
7. Minh BQ, Schmidt HA, Chernomor O, Schrempf D, Woodhams MD, von Haeseler A, et al. IQ-TREE 2: New Models and Efficient Methods for Phylogenetic Inference in the Genomic Era. Molecular Biology and Evolution. 2020 May 1;37(5):1530–4.
8. Hoang DT, Chernomor O, von Haeseler A, Minh BQ, Vinh LS. UFBoot2: Improving the Ultrafast Bootstrap Approximation. Molecular Biology and Evolution. 2018 Feb 1;35(2):518–22.
9. Moody ERR, Mahendrarajah TA, Dombrowski N, Clark JW, Petitjean C, Offre P, et al. Universal markers support a long inter-domain branch between Archaea and Bacteria. bioRxiv. 2021 Jan 20;2021.01.19.427276.
10. Gouy R, Baurain D, Philippe H. Rooting the tree of life: the phylogenetic jury is still out. Philosophical Transactions of the Royal Society B: Biological Sciences. 2015 Sep 26;370(1678):20140329.
11. Jones DT, Taylor WR, Thornton JM. A mutation data matrix for transmembrane proteins. FEBS Letters. 1994 Feb 21;339(3):269–75.
