# Supplementary material for: A unified evolutionary origin for the ubiquitous protein transporters SecY and YidC
Source: BMC Biol. 2021 Dec 15;19:266. doi: 10.1186/s12915-021-01171-5 (PMC8675477; doi:10.1186/s12915-021-01171-5)
Supplement: Supplementary file 2 — Additional file 2. Figure S1. Structural similarity and symmetry breaking between SecY halves. Figure S2. Structures of non-Oxa1 superfamily top Dali hits. Figure S3. Amino acid conservation in bacterial YidC. Figure S4. Structure and contact prediction for archaeal and human heterodimers homologous to EMC3/6. Figure S5. Similarity between archaeal and bacterial SecG. [file 12915_2021_1171_MOESM2_ESM.pdf]

a Structural similarity (TM-score)

Taxon	State	C-half	N-half							
			5aww	3din	**6itc**	7kb5	**6fti**	3jc2	1rh5	mean
Bacteria	Closed	5aww	0.67	0.60	0.62	0.68	0.71	0.68	0.68	0.66
	Primed	3din	0.65	0.64	0.62	0.65	0.62	0.65	0.67	0.64
	Open	**6itc**	0.74	0.69	**0.75**	0.77	**0.81**	0.77	0.77	0.76
Eukarya	Closed	7kb5	0.68	0.68	0.68	0.66	0.71	0.66	0.68	0.68
	Primed	6fti	0.68	0.67	0.67	0.67	0.72	0.67	0.69	0.68
	Open	3jc2	0.69	0.68	0.68	0.68	0.72	0.70	0.70	0.69
Archaea	Closed	1rh5	0.67	0.64	0.64	0.65	0.70	0.65	0.66	0.66
		mean	0.68	0.66	0.67	0.68	0.71	0.68	0.69	

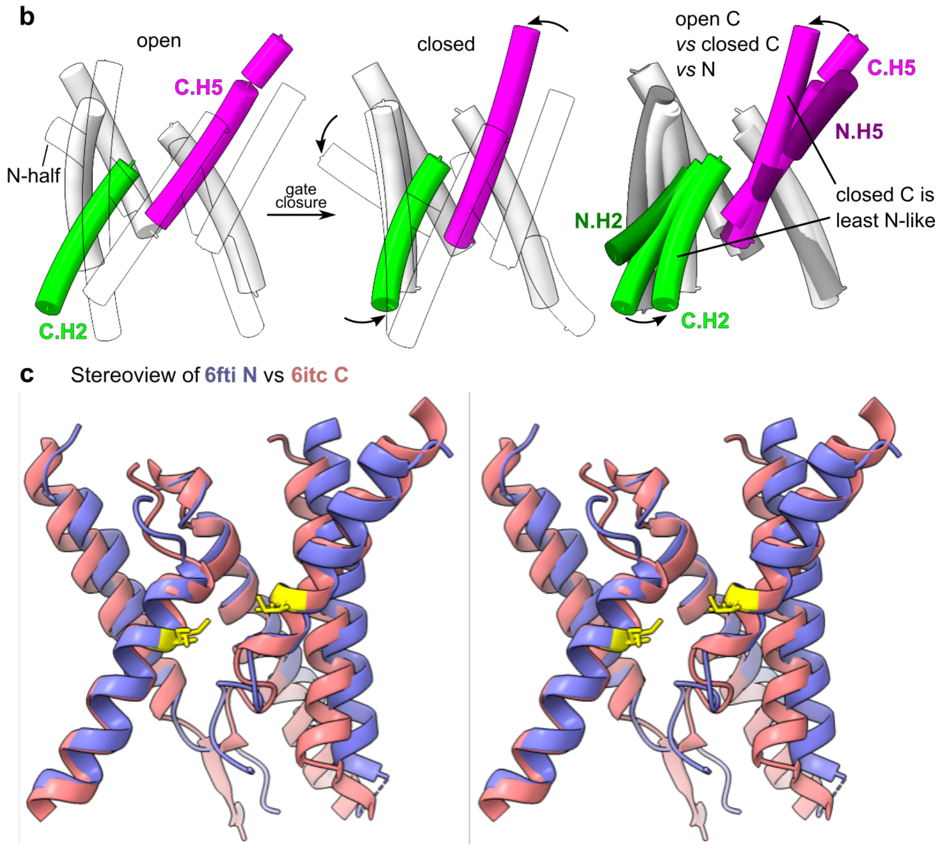

Figure S1: Structural similarity and symmetry breaking between SecY halves. **a** Pairwise comparisons of the SecY halves by TM-score. TM-scores can range from 0 to 1. Boldface scores indicate the most similar pair from the same structure (6itc) or from any structure (6itc C, 6fti N). **b** Symmetry-breaking tilts in C.H2 and C.H5 induced by channel closure. At left, the TMHs from open (5aww) and closed (6itc) models are shown, with the N-half rendered in transparent outline. At right is a superposition of closed (5aww) and open C-halves (6itc) and an N-half (6itc). All models were aligned by H1/4. **c** Walleye stereoview of the most similar pair of N- and C-halves. The pore ring residues are shown as sticks and highlighted yellow.

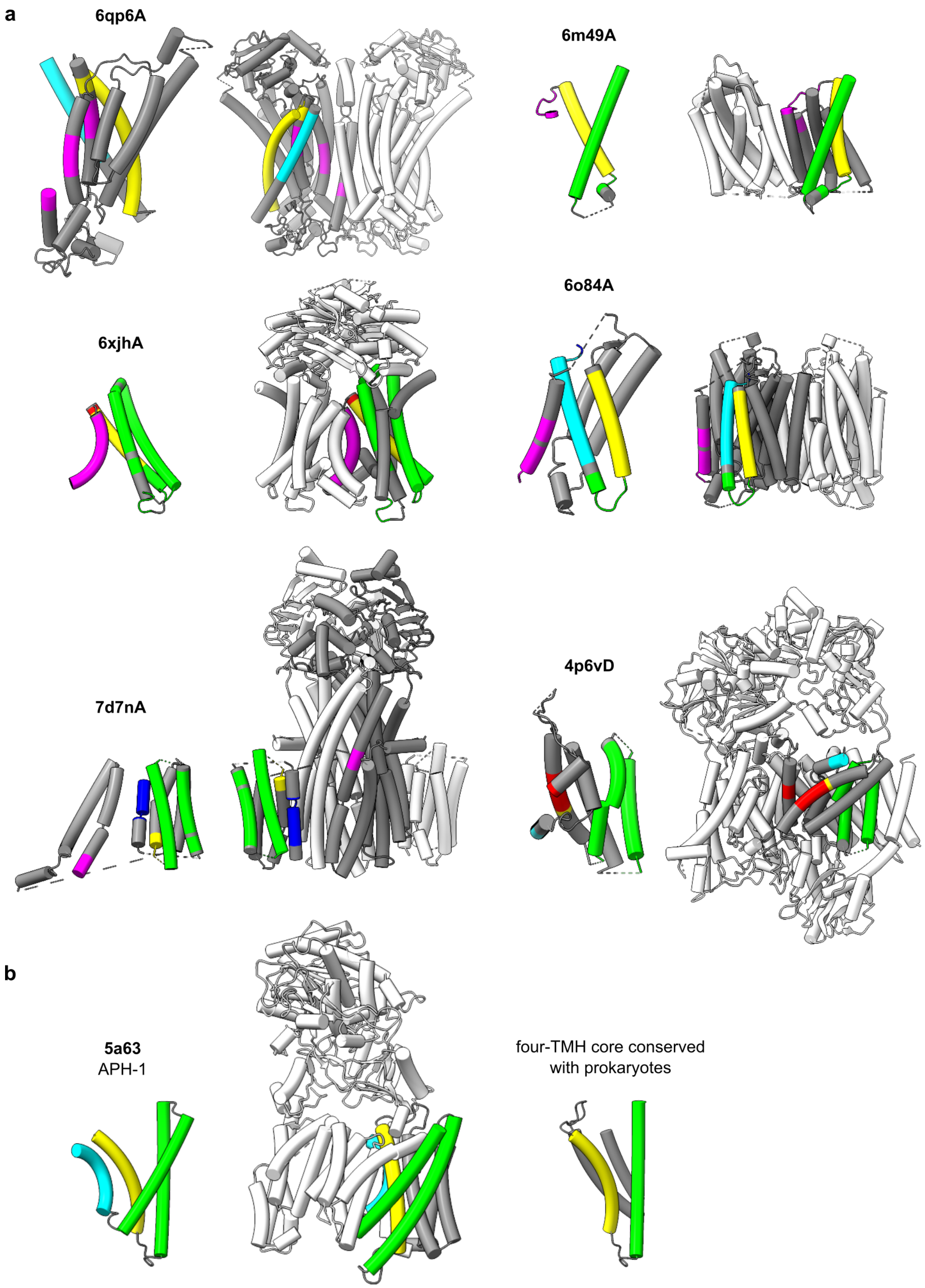

Figure S2: Structures of non-Oxa1 superfamily top Dali hits. **a** Isolated hits. The aligned chain is shown in grey, and any other chains in the model are shown in white. Regions of each hit are colour-coded according to which part of the query structure they aligned with, using the colour scheme from Figure 4. **b** The multiple hit APH-1. In addition to the representations shown in panel a, at right are shown the four TMHs conserved among APH-1's prokaryotic homologs. As in a, the non-aligned TMHs are shown in grey, and the two aligned TMHs are coloured.

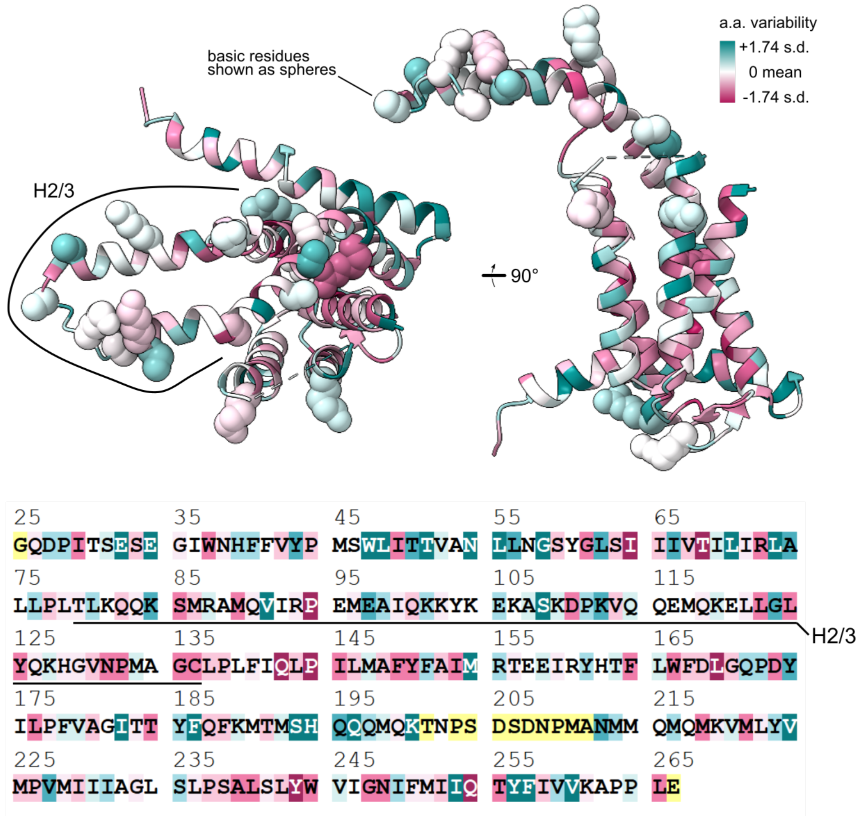

Figure S3: Amino acid conservation in bacterial YidC. The conservation scores and annotated sequence and PDB model shown (*B. halodurans* YidC2, 3wo7A) were retrieved from ConSurf-DB. The structure is colour-coded using the continuous scale shown, whereas the sequence is colour-coded on a similar but quantized scale. The scale shown encompasses the minimum but not maximum score.

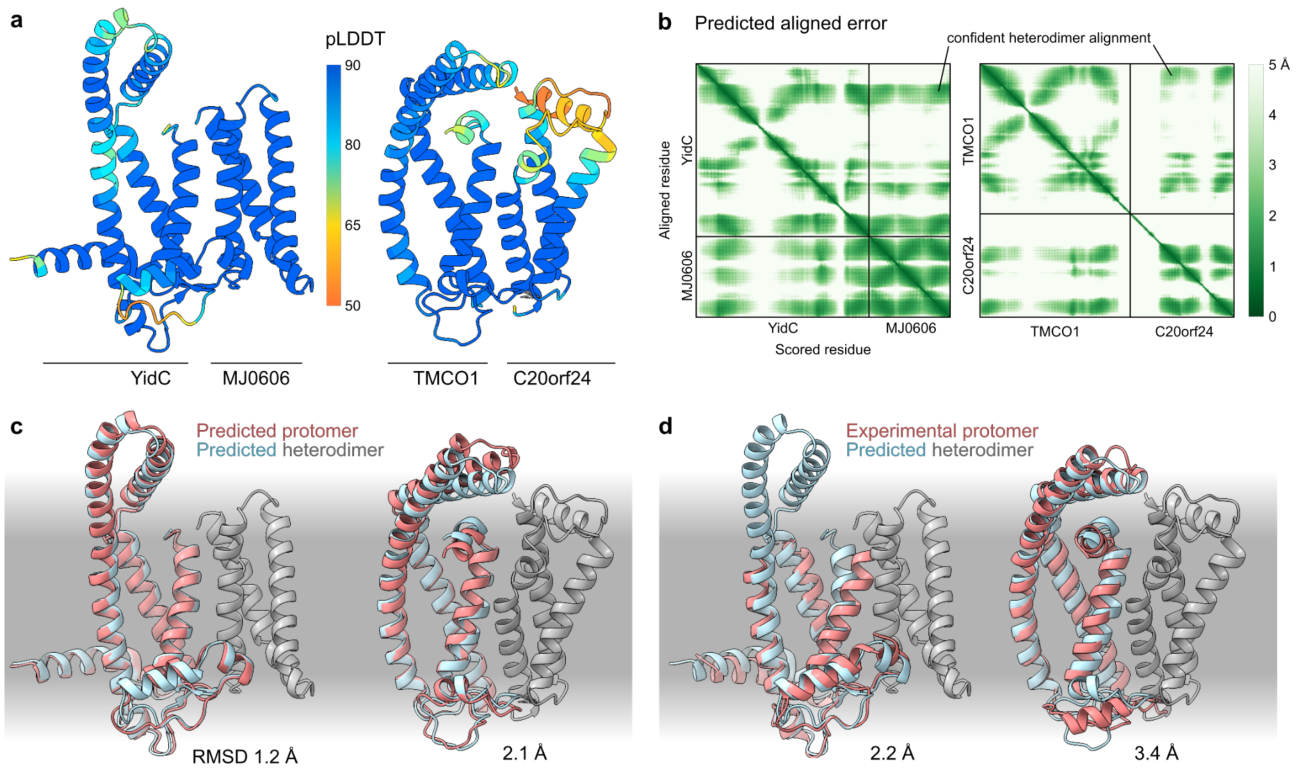

Figure S4: Structure and contact prediction for archaeal and human heterodimers homologous to EMC3/6. **a** Per-residue confidence scores (predicted local distance difference test, pLDDT) for the YidC a.k.a. MJ0480/MJ0606 and TMCO1/C20orf24 heterodimers. **b** Predicted aligned error. **c** Alignments showing the predicted structure for each heterodimer (light blue, grey) *vs* the predicted structure for the Oxa1 superfamily protomer alone (coral). **d** As in panel c except aligned *vs* experimentally determined structures for the Oxa1 superfamily protomer alone (YidC: 5c8j, TMCO1: 6w6l).

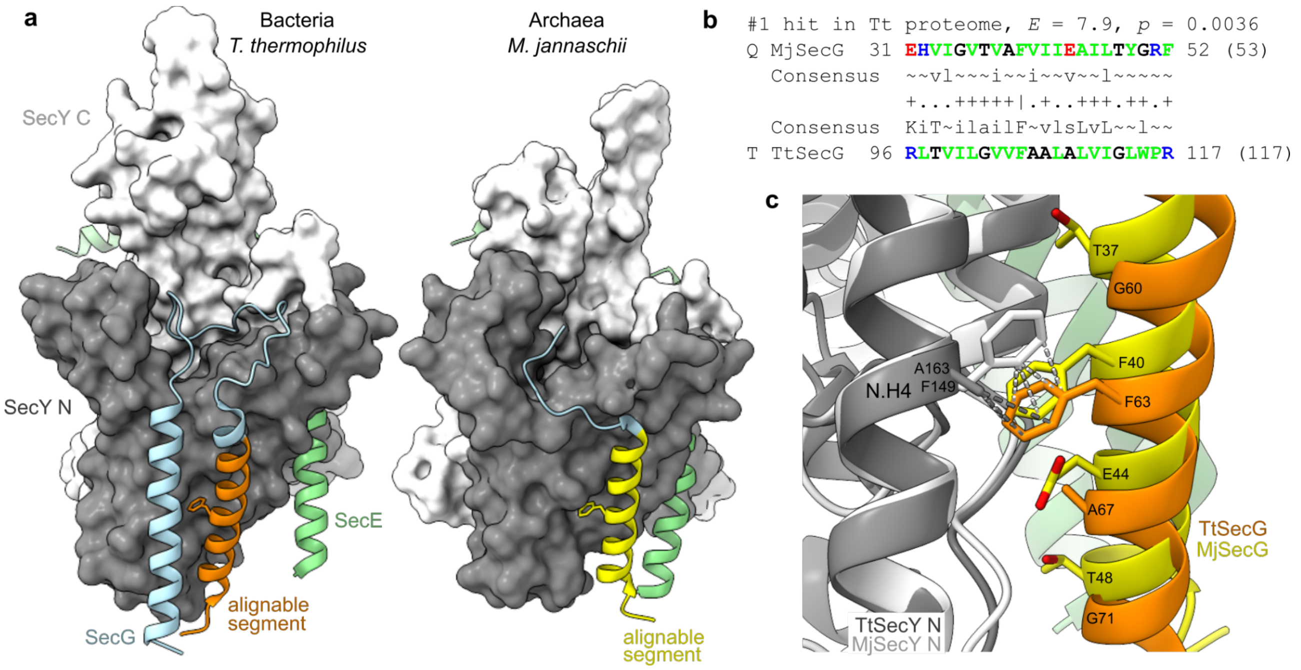

Figure S5. Similarity between archaeal and bacterial SecG. **a** *T. thermophilus* and *M. jannaschii* SecYEG, shown separately but oriented by aligning the two SecY TMs against which SecG packs (N.H1 and N.H4). The SecG subsequences identified as homologous by HHpred (panel **b**) are highlighted in orange and yellow. **b** The top hit from an HHpred query of the *T. thermophilus* proteome with *M. jannaschii* SecG. **c** The hydrophilic face of SecG and a conserved phenylalanine which contacts a specific residue in SecY N.H4.
