# Supplementary figures and images for: A unified evolutionary origin for the ubiquitous protein transporters SecY and YidC
Source: BMC Biol. 2021 Dec 15;19:266. doi: 10.1186/s12915-021-01171-5 (PMC8675477; doi:10.1186/s12915-021-01171-5)

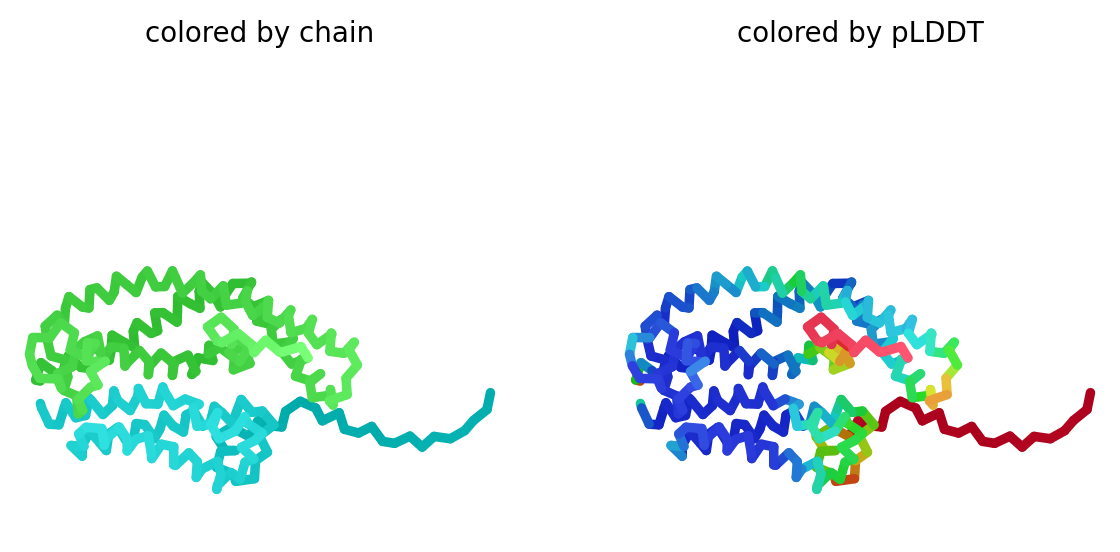

Supplement: Supplementary file 5 — Additional file 5. Predicted MJ0480/MJ0606 and TMCO1/C02orf24 structures. [file 12915_2021_1171_MOESM5_ESM.zip › Additional file 5. Predicted structures/prediction_TMCO1iso3_C20orf24iso2/rank_4_model_1_ptm_seed_0.png]

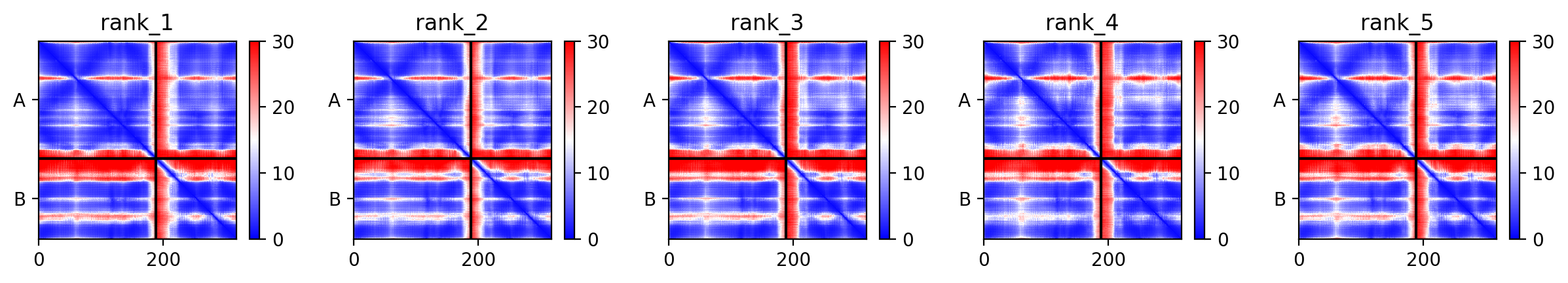

Supplement: Supplementary file 5 — Additional file 5. Predicted MJ0480/MJ0606 and TMCO1/C02orf24 structures. [file 12915_2021_1171_MOESM5_ESM.zip › Additional file 5. Predicted structures/prediction_TMCO1iso3_C20orf24iso2/predicted_alignment_error.png]

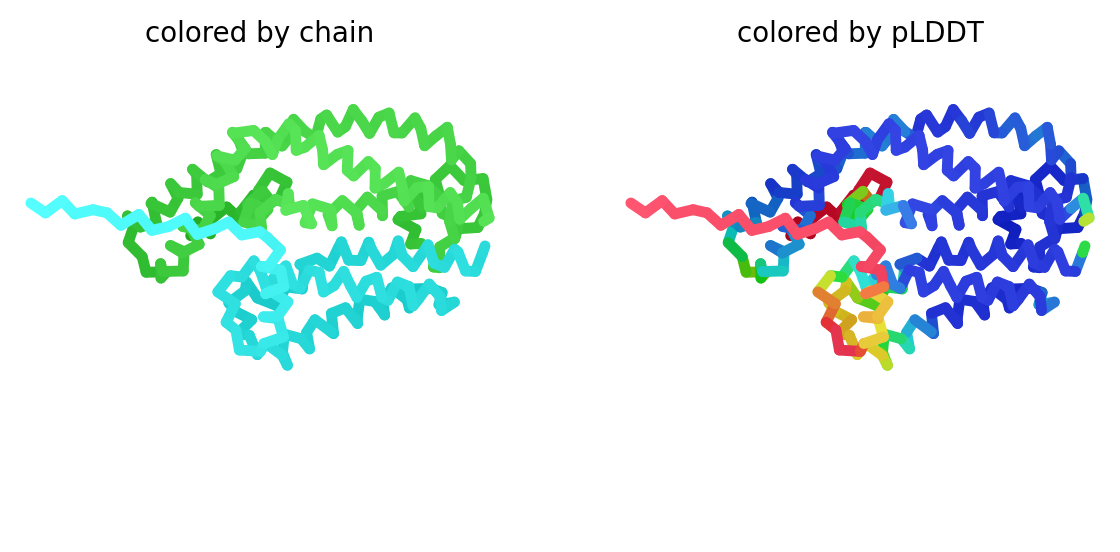

Supplement: Supplementary file 5 — Additional file 5. Predicted MJ0480/MJ0606 and TMCO1/C02orf24 structures. [file 12915_2021_1171_MOESM5_ESM.zip › Additional file 5. Predicted structures/prediction_TMCO1iso3_C20orf24iso2/rank_1_model_5_ptm_seed_0.png]

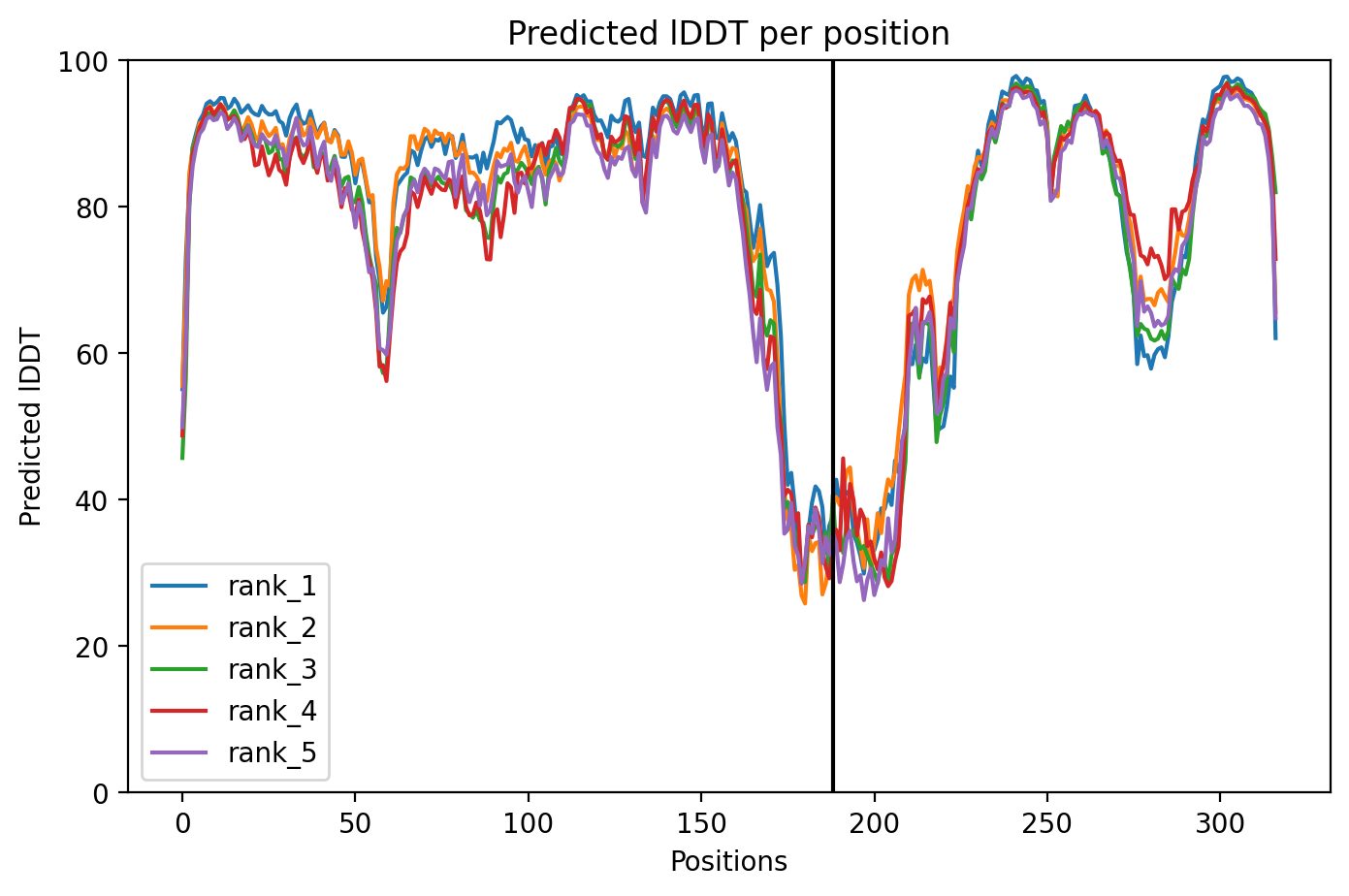

Supplement: Supplementary file 5 — Additional file 5. Predicted MJ0480/MJ0606 and TMCO1/C02orf24 structures. [file 12915_2021_1171_MOESM5_ESM.zip › Additional file 5. Predicted structures/prediction_TMCO1iso3_C20orf24iso2/predicted_LDDT.png]

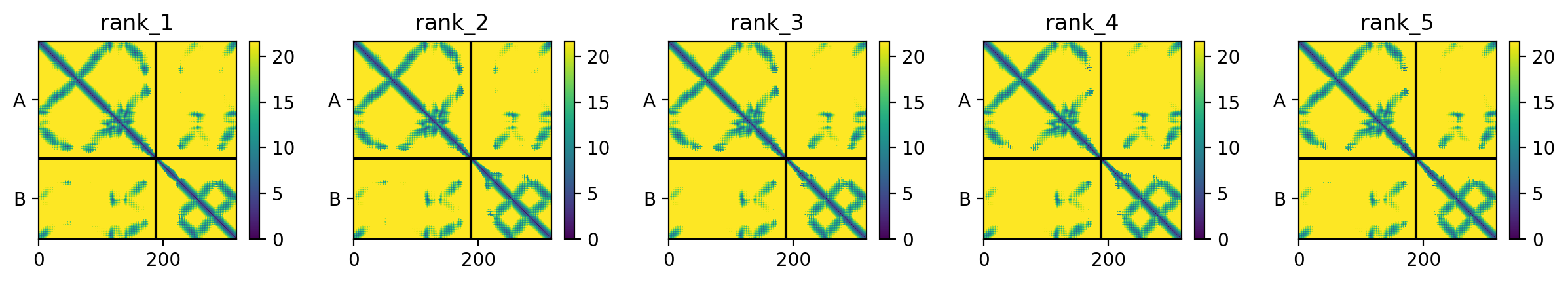

Supplement: Supplementary file 5 — Additional file 5. Predicted MJ0480/MJ0606 and TMCO1/C02orf24 structures. [file 12915_2021_1171_MOESM5_ESM.zip › Additional file 5. Predicted structures/prediction_TMCO1iso3_C20orf24iso2/predicted_distogram.png]

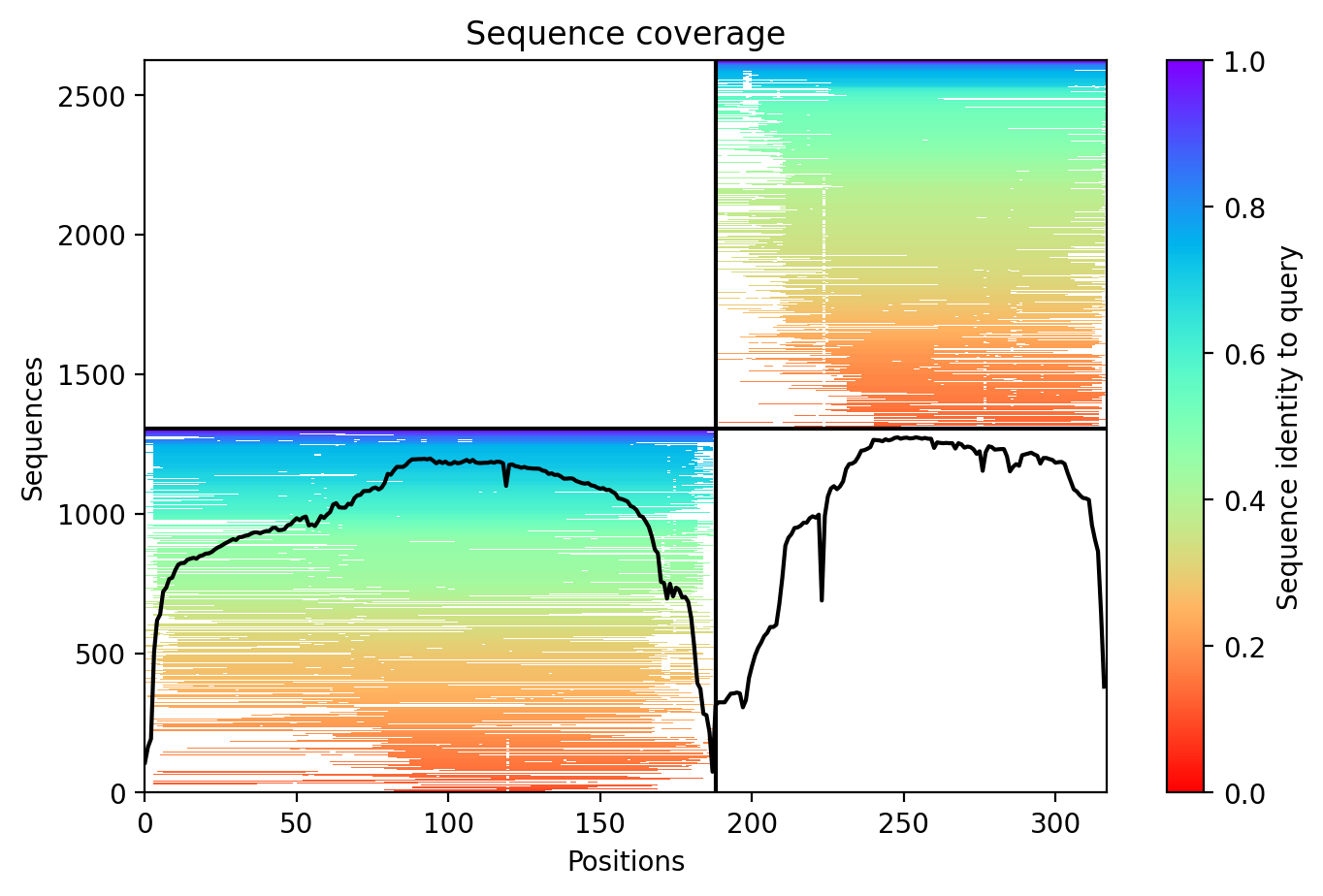

Supplement: Supplementary file 5 — Additional file 5. Predicted MJ0480/MJ0606 and TMCO1/C02orf24 structures. [file 12915_2021_1171_MOESM5_ESM.zip › Additional file 5. Predicted structures/prediction_TMCO1iso3_C20orf24iso2/msa_coverage.png]

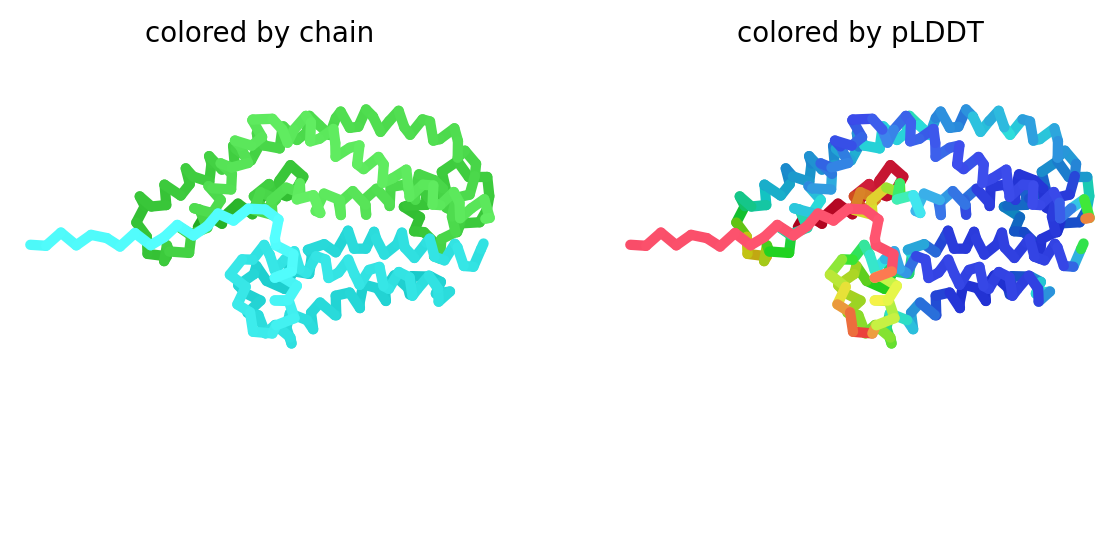

Supplement: Supplementary file 5 — Additional file 5. Predicted MJ0480/MJ0606 and TMCO1/C02orf24 structures. [file 12915_2021_1171_MOESM5_ESM.zip › Additional file 5. Predicted structures/prediction_TMCO1iso3_C20orf24iso2/rank_5_model_4_ptm_seed_0.png]

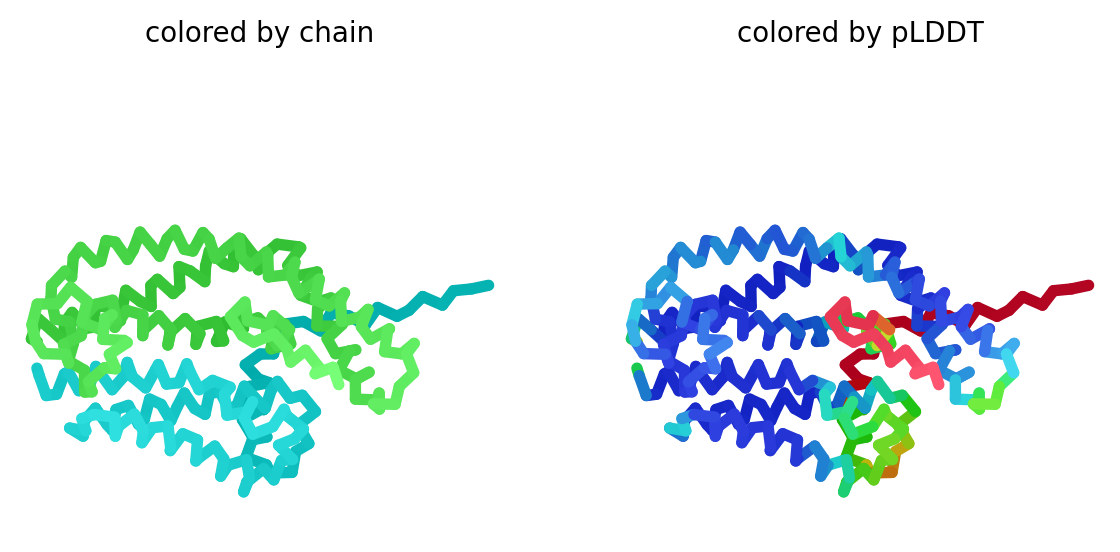

Supplement: Supplementary file 5 — Additional file 5. Predicted MJ0480/MJ0606 and TMCO1/C02orf24 structures. [file 12915_2021_1171_MOESM5_ESM.zip › Additional file 5. Predicted structures/prediction_TMCO1iso3_C20orf24iso2/rank_2_model_3_ptm_seed_0.png]

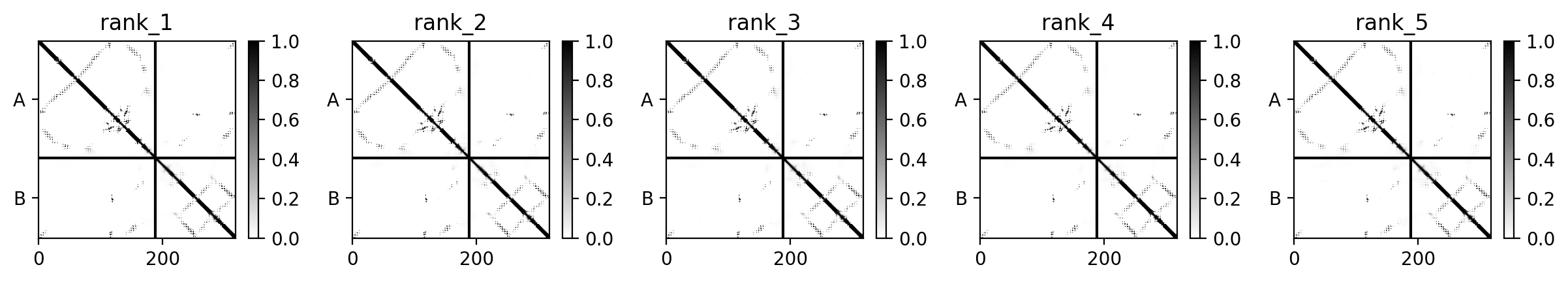

Supplement: Supplementary file 5 — Additional file 5. Predicted MJ0480/MJ0606 and TMCO1/C02orf24 structures. [file 12915_2021_1171_MOESM5_ESM.zip › Additional file 5. Predicted structures/prediction_TMCO1iso3_C20orf24iso2/predicted_contacts.png]

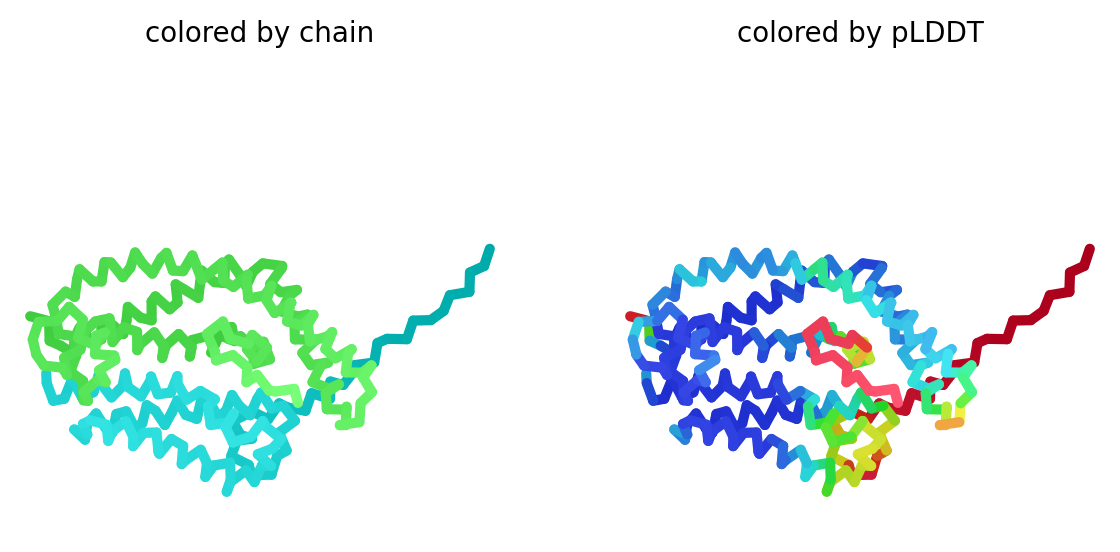

Supplement: Supplementary file 5 — Additional file 5. Predicted MJ0480/MJ0606 and TMCO1/C02orf24 structures. [file 12915_2021_1171_MOESM5_ESM.zip › Additional file 5. Predicted structures/prediction_TMCO1iso3_C20orf24iso2/rank_3_model_2_ptm_seed_0.png]

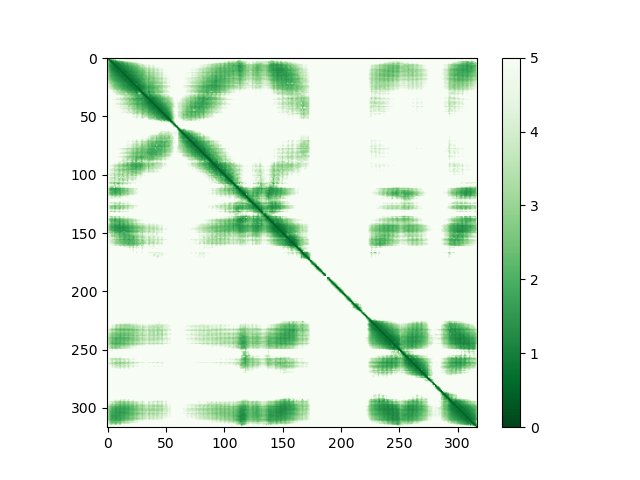

Supplement: Supplementary file 5 — Additional file 5. Predicted MJ0480/MJ0606 and TMCO1/C02orf24 structures. [file 12915_2021_1171_MOESM5_ESM.zip › Additional file 5. Predicted structures/prediction_TMCO1iso3_C20orf24iso2/rank_1_custom_pae.png]

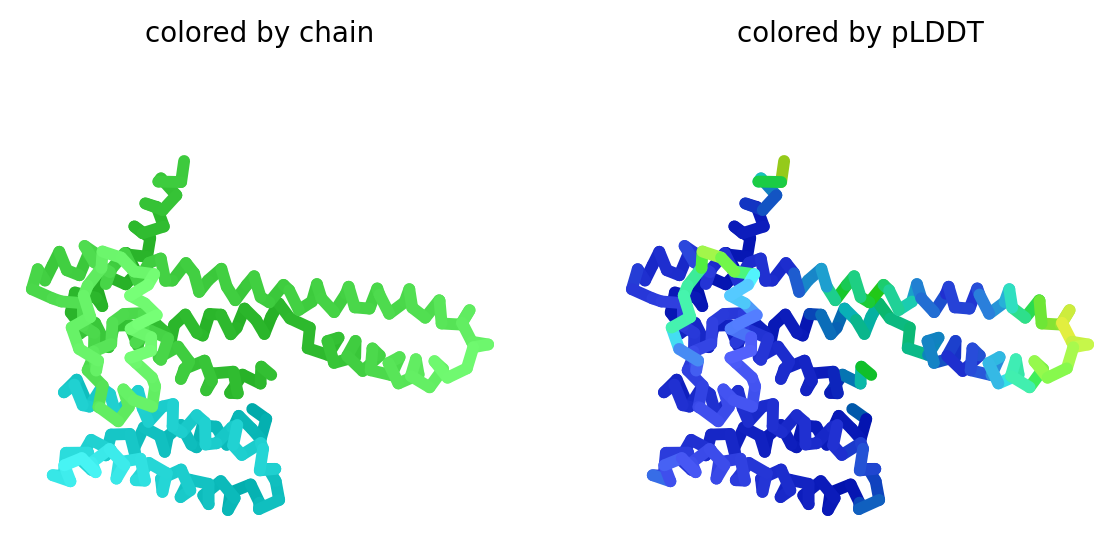

Supplement: Supplementary file 5 — Additional file 5. Predicted MJ0480/MJ0606 and TMCO1/C02orf24 structures. [file 12915_2021_1171_MOESM5_ESM.zip › Additional file 5. Predicted structures/prediction_MJ0480_MJ0606/rank_4_model_1_ptm_seed_0.png]

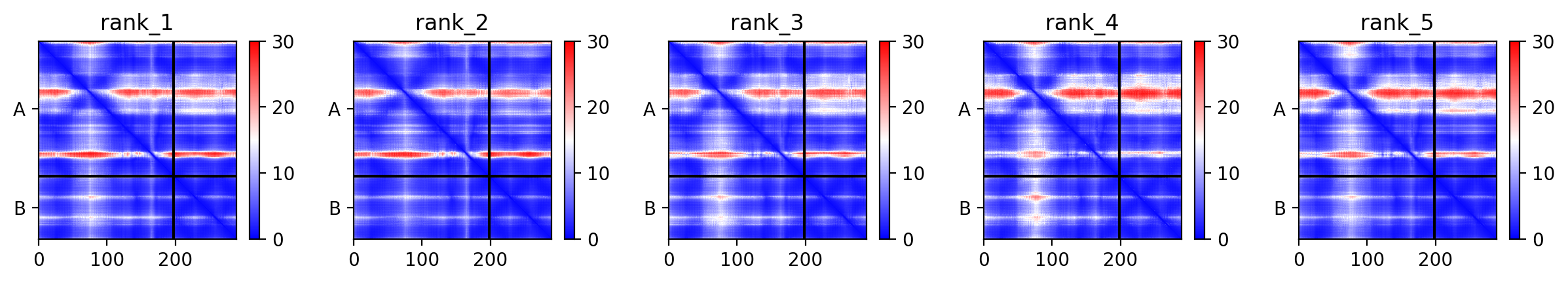

Supplement: Supplementary file 5 — Additional file 5. Predicted MJ0480/MJ0606 and TMCO1/C02orf24 structures. [file 12915_2021_1171_MOESM5_ESM.zip › Additional file 5. Predicted structures/prediction_MJ0480_MJ0606/predicted_alignment_error.png]

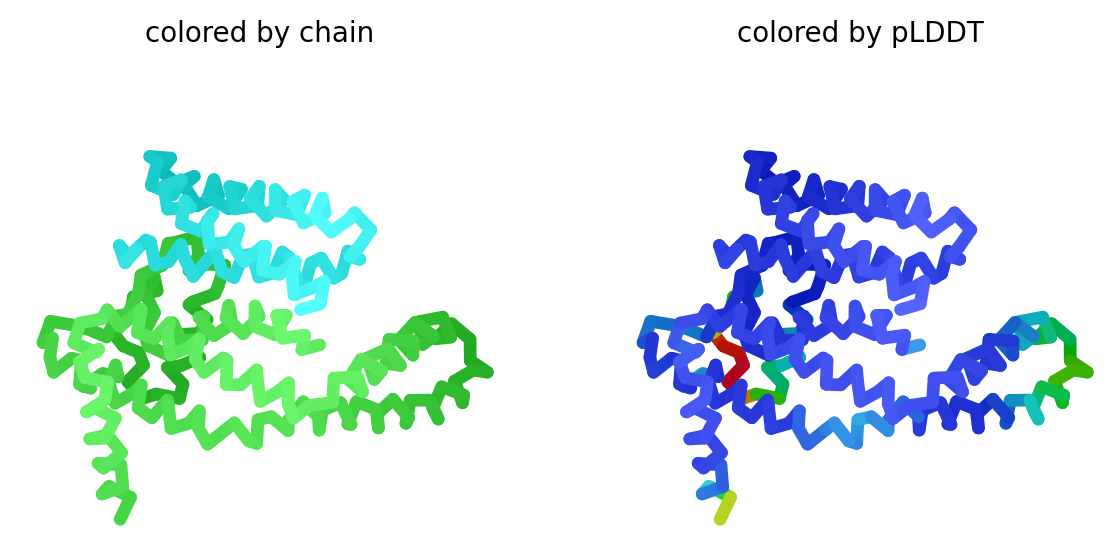

Supplement: Supplementary file 5 — Additional file 5. Predicted MJ0480/MJ0606 and TMCO1/C02orf24 structures. [file 12915_2021_1171_MOESM5_ESM.zip › Additional file 5. Predicted structures/prediction_MJ0480_MJ0606/rank_2_model_4_ptm_seed_0.png]

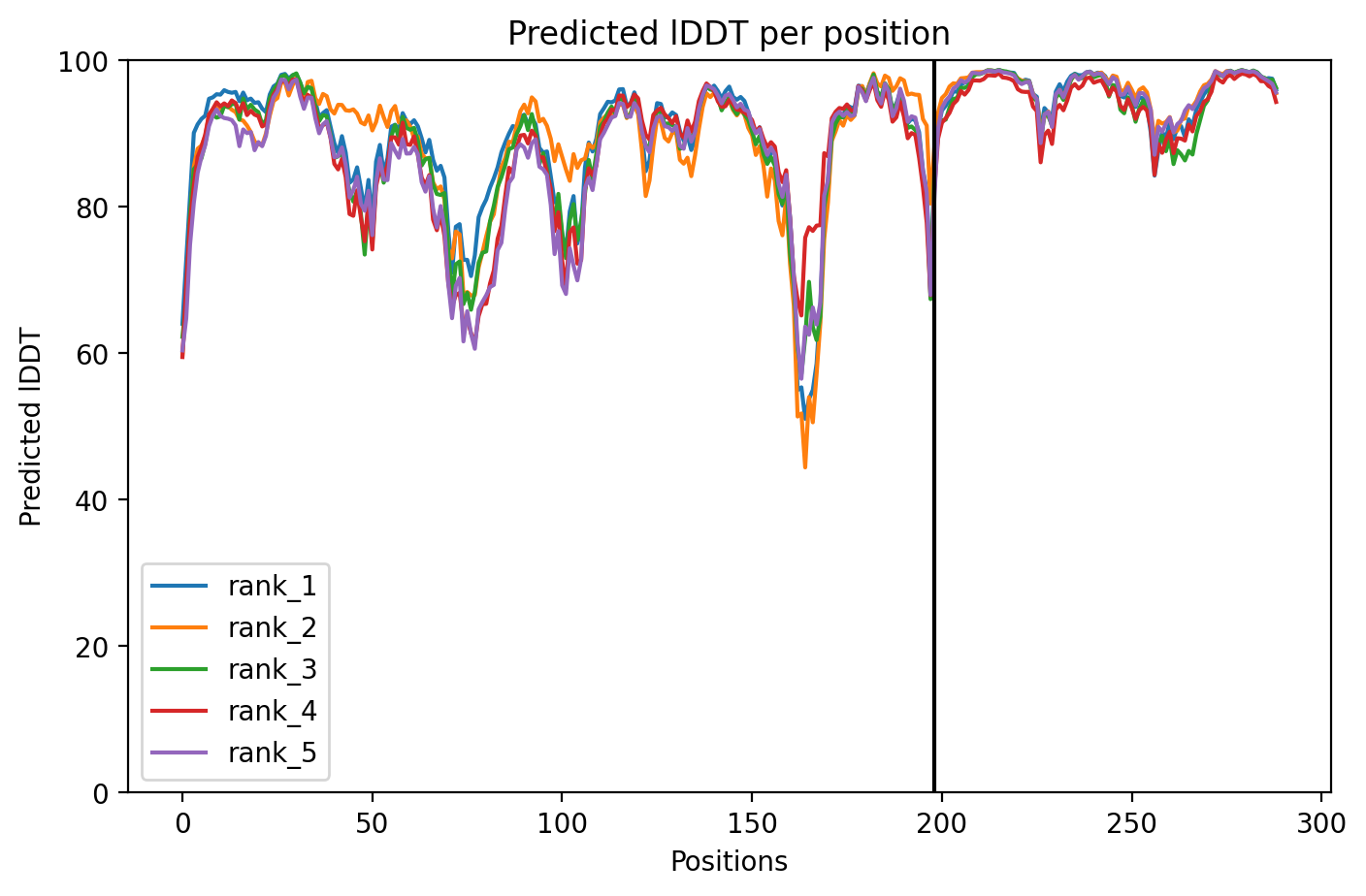

Supplement: Supplementary file 5 — Additional file 5. Predicted MJ0480/MJ0606 and TMCO1/C02orf24 structures. [file 12915_2021_1171_MOESM5_ESM.zip › Additional file 5. Predicted structures/prediction_MJ0480_MJ0606/predicted_LDDT.png]

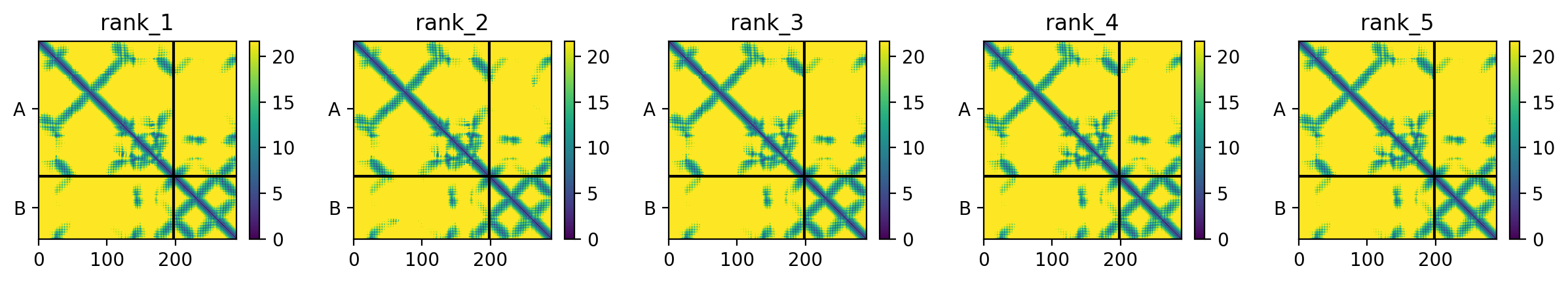

Supplement: Supplementary file 5 — Additional file 5. Predicted MJ0480/MJ0606 and TMCO1/C02orf24 structures. [file 12915_2021_1171_MOESM5_ESM.zip › Additional file 5. Predicted structures/prediction_MJ0480_MJ0606/predicted_distogram.png]

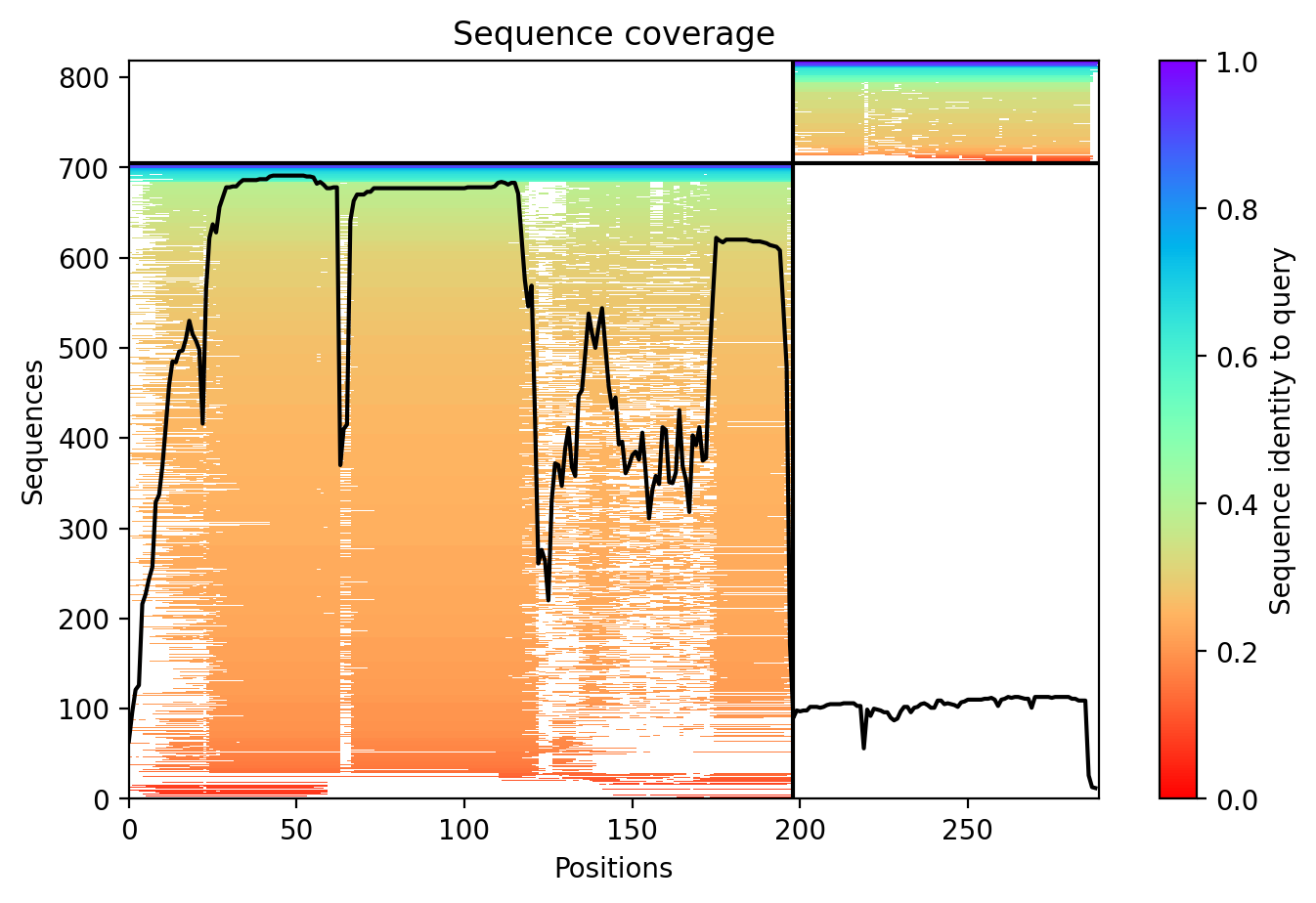

Supplement: Supplementary file 5 — Additional file 5. Predicted MJ0480/MJ0606 and TMCO1/C02orf24 structures. [file 12915_2021_1171_MOESM5_ESM.zip › Additional file 5. Predicted structures/prediction_MJ0480_MJ0606/msa_coverage.png]

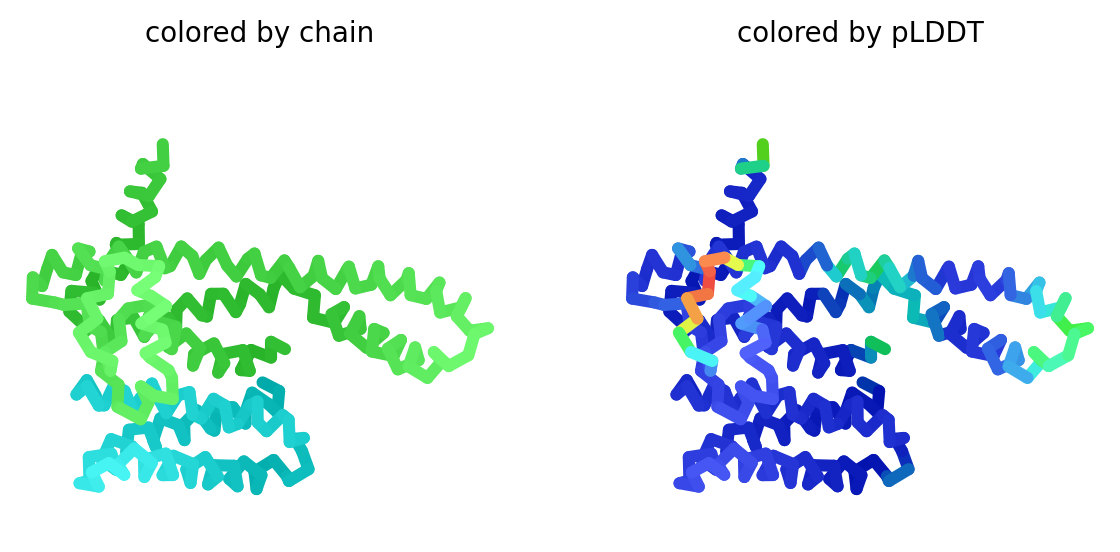

Supplement: Supplementary file 5 — Additional file 5. Predicted MJ0480/MJ0606 and TMCO1/C02orf24 structures. [file 12915_2021_1171_MOESM5_ESM.zip › Additional file 5. Predicted structures/prediction_MJ0480_MJ0606/rank_1_model_3_ptm_seed_0.png]

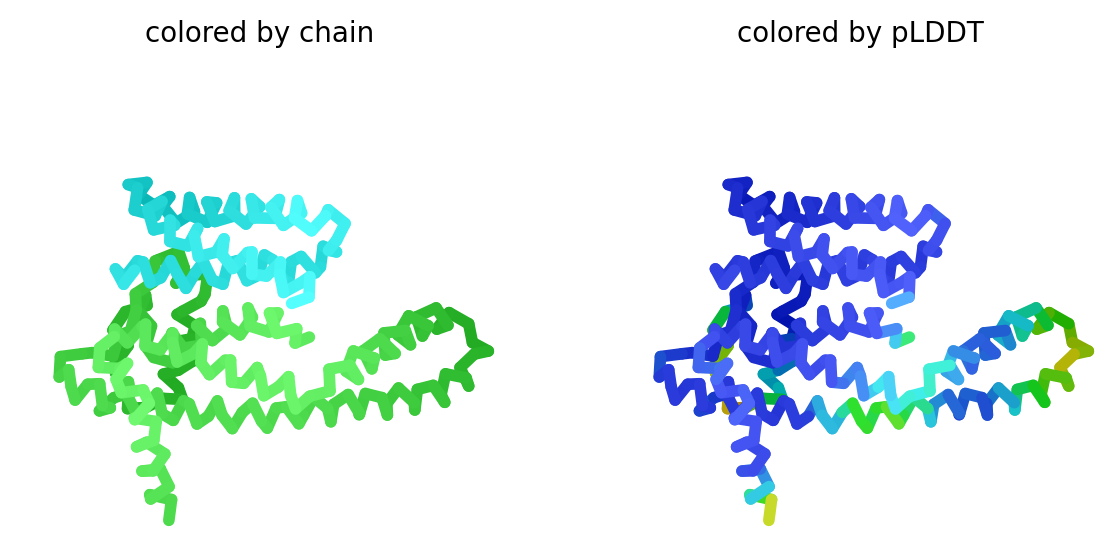

Supplement: Supplementary file 5 — Additional file 5. Predicted MJ0480/MJ0606 and TMCO1/C02orf24 structures. [file 12915_2021_1171_MOESM5_ESM.zip › Additional file 5. Predicted structures/prediction_MJ0480_MJ0606/rank_5_model_2_ptm_seed_0.png]

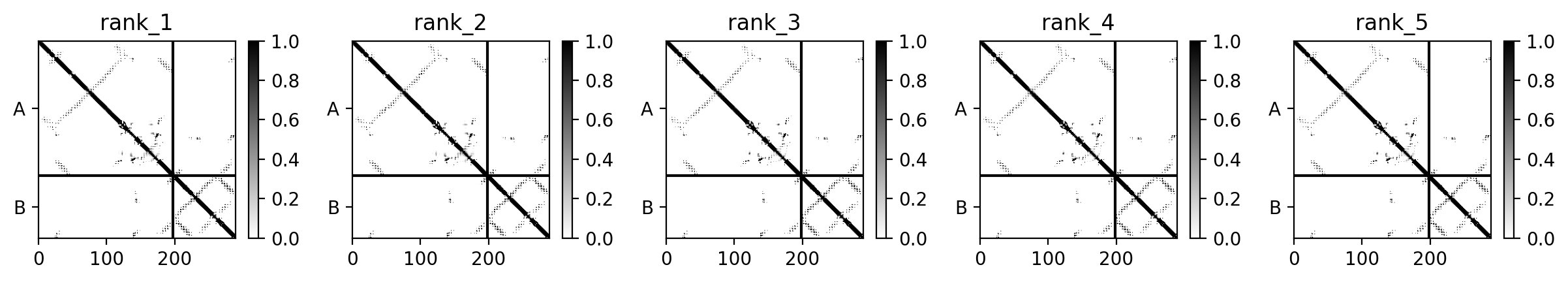

Supplement: Supplementary file 5 — Additional file 5. Predicted MJ0480/MJ0606 and TMCO1/C02orf24 structures. [file 12915_2021_1171_MOESM5_ESM.zip › Additional file 5. Predicted structures/prediction_MJ0480_MJ0606/predicted_contacts.png]

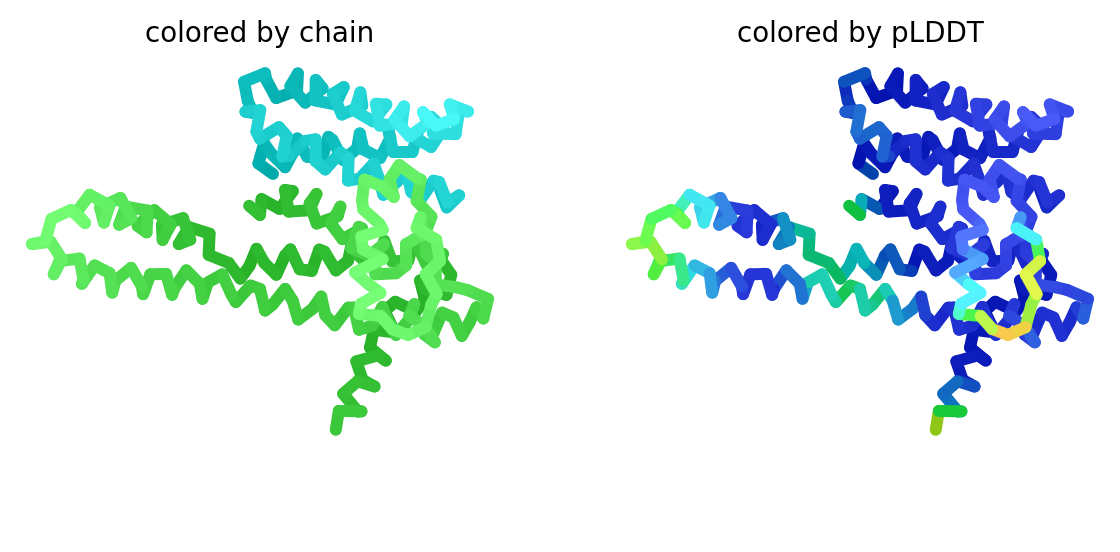

Supplement: Supplementary file 5 — Additional file 5. Predicted MJ0480/MJ0606 and TMCO1/C02orf24 structures. [file 12915_2021_1171_MOESM5_ESM.zip › Additional file 5. Predicted structures/prediction_MJ0480_MJ0606/rank_3_model_5_ptm_seed_0.png]

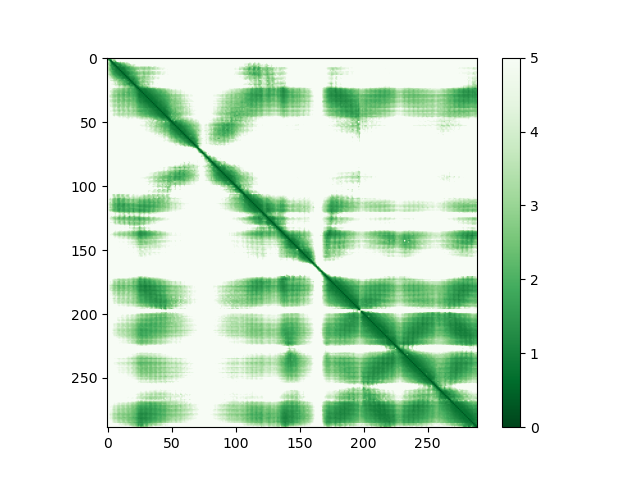

Supplement: Supplementary file 5 — Additional file 5. Predicted MJ0480/MJ0606 and TMCO1/C02orf24 structures. [file 12915_2021_1171_MOESM5_ESM.zip › Additional file 5. Predicted structures/prediction_MJ0480_MJ0606/rank_1_custom_pae.png]
